# Approaches to Quality Risk Management When Using Single-Use Systems in the Manufacture of Biologics

**DOI:** 10.1208/s12249-015-0368-z

**Published:** 2015-08-20

**Authors:** Akiko Ishii-Watabe, Akihiko Hirose, Noriko Katori, Norikata Hashii, Susumu Arai, Hirotoshi Awatsu, Akira Eiza, Yoshiaki Hara, Hideshi Hattori, Tomomi Inoue, Tetsuya Isono, Masahiro Iwakura, Daisuke Kajihara, Nobuo Kasahara, Hiroyuki Matsuda, Sei Murakami, Taishiro Nakagawa, Takehiro Okumura, Takeshi Omasa, Shinya Takuma, Iyo Terashima, Masayoshi Tsukahara, Maiko Tsutsui, Takahiro Yano, Nana Kawasaki

**Affiliations:** National Institute of Health Sciences, 1-18-1 Kamiyoga, Setagaya-ku, Tokyo, 158-8501 Japan; Sumitomo Bakelite Co., Ltd., 1-5, Murotani 1-Chome, Nishi-ku, Kobe, 651-2241 Japan; Nihon Pall Ltd., 6-5-1, Nishishinjuku, Shinjuku-ku, Tokyo, 163-1325 Japan; Sekisui Seikei, Ltd., 2-1-9 Dojimahama, Kita-ku, Osaka, 530-0004 Japan; Sartorius Stedim Japan, 1-8-11 Kitashinagawa Shinagawa-ku, Tokyo, 140-0001 Japan; Dai Nippon Printing Co., Ltd., 1-1-1, Ichigaya Kagacho, Shinjuku-ku, Tokyo, 162-8001 Japan; MSD K.K., 2-3-7 Hiranomachi, Chuo-ku, Osaka, 541-0046 Japan; Chugai Pharmaceutical Co., Ltd., 5-1 Ukima 5-Chome Kita-ku, Tokyo, 115-8543 Japan; Manufacturing Technology Association of Biologics, 2-1-10 Shinkawa, Chuo-ku, Tokyo, 104-0033 Japan; GE Healthcare Japan Corporation, Sanken Bldg., 3-25-1 Hyakuninchio, Shinjuku-ku, Tokyo, 169-0073 Japan; Astellas Pharma Tech Co., Ltd., 160-2 Akahama, Takahagi-shi, Ibaraki, 318-0001 Japan; Fujimori Kogyo Co., Ltd, 1-23-7 Nishi-Shinjuku, Shinjuku-ku, Tokyo, 160-0023 Japan; Hitachi, Ltd., 4-5-2 Higashi-Ikebukuro, Toshima-ku, Tokyo, 170-8466 Japan; Kyowa Hakko Kirin Co., Ltd., 1-6-1 Ohtemachi, Chiyoda-ku, Tokyo, 100-8185 Japan; Takeda Pharmaceutical Co., Ltd., 17-85, Jusohonmachi 2-chome, Yodogawa-ku, Osaka, 532-8686 Japan; Osaka University, U1E801, 2-1 Yamadaoka, Suita-shi, Osaka, 565-0871 Japan; Merck Millipore, 1-8-1 Shimomeguro, Meguro-ku, Tokyo, 153-8927 Japan; Sumitomo Dainippon Pharma Co., Ltd., 1-3-45 Kurakakiuchi, Ibaraki-shi, Osaka, 567-0878 Japan; Daiichi Sankyo Co., Ltd., 1-12-1, Shinomiya, Hiratsuka-shi, Kanagawa, 254-0014 Japan; Yokohama City University, 1-7-29 Suehirocho, Tsurumi-ku, Yokohama-shi, Kanagawa, 230-0045 Japan

**Keywords:** biologics, manufacturing technology, quality risk management, regulatory science, single-use system

## Abstract

Biologics manufacturing technology has made great progress in the last decade. One of the most promising new technologies is the single-use system, which has improved the efficiency of biologics manufacturing processes. To ensure safety of biologics when employing such single-use systems in the manufacturing process, various issues need to be considered including possible extractables/leachables and particles arising from the components used in single-use systems. Japanese pharmaceutical manufacturers, together with single-use suppliers, members of the academia and regulatory authorities have discussed the risks of using single-use systems and established control strategies for the quality assurance of biologics. In this study, we describe approaches for quality risk management when employing single-use systems in the manufacturing of biologics. We consider the potential impact of impurities related to single-use components on drug safety and the potential impact of the single-use system on other critical quality attributes as well as the stable supply of biologics. We also suggest a risk-mitigating strategy combining multiple control methods which includes the selection of appropriate single-use components, their inspections upon receipt and before releasing for use and qualification of single-use systems. Communication between suppliers of single-use systems and the users, as well as change controls in the facilities both of suppliers and users, are also important in risk-mitigating strategies. Implementing these control strategies can mitigate the risks attributed to the use of single-use systems. This study will be useful in promoting the development of biologics as well as in ensuring their safety, quality and stable supply.

## INTRODUCTION

A single-use system is a system comprised of single-use components, as the main apparatus/equipment. In recent years, the use of single-use systems, such as single-use culture bags in place of the conventional stainless steel culture tanks, has been increasing in the manufacture of biologics (i.e. recombinant therapeutic proteins). Utilization of novel single-use technologies has promising advantages that include increased efficiency of manufacturing, including prevention of cross contamination in the manufacture of high-potency compounds, flexibility to manufacture multiple products and elimination of the need for cleaning and steam sterilization including those validations. However, this approach has also raised new issues and concerns related to process control and product quality assurance.

Based on accumulated scientific knowledge and quality risk management concepts, this study identifies points that need to be considered with respect to quality assurance for biologics manufactured with single-use systems. The issues described to ensure the quality of biologics manufactured using single-use systems reflect generally accepted concepts, but the individual risks and the development of a risk-mitigating strategy should not be limited to these. In each individual case, aspects raised in this study may be applied selectively, but it is of paramount importance that the risks are appropriately assessed according to technological advances and the quality profile of the intended product and that appropriate risk control is implemented correctly.

## SCOPE

This study applies to single-use components, including bags, filters, tubing, and connectors used in processes for manufacturing biologic drug substance and drug products, as well as systems comprised of single-use components. Drug product containers (primary packaging), however, are outside the scope of this study, since in these cases, the appropriate requirements are stipulated by the pharmacopoeia (e.g. *Japanese Pharmacopoeia*).

## BASIC REQUIREMENTS

The quality assurance of biologics encompasses an evaluation of the raw materials as well as manufacturing process controls for the drug substance and drug product. All procedures must comply with ministerial regulation of good manufacturing practice and the pharmacopoeia (e.g. *Japanese Pharmacopoeia*) [1–15], in addition to other regional requirements [16–20], such as the Standard for Biological Ingredients in Japan. ICH Q9 and other relevant guidelines [21–32] are useful references for the general rules and procedures recommended for quality risk management. Identification of the risks that affect quality, efficacy, safety and stable supply of biologics and the establishment of control strategies are basic requirements for the development of a manufacturing process of biologics using single-use systems. These risk management processes should be shared between the relevant parties.

There are important elements in quality systems that are intended to improve the quality and stable supply of biologics. Establishing quality control strategies on the basis of appropriate risk assessments is beneficial to ongoing improvement throughout the biologics lifecycle, as well as communication between single-use component suppliers, end users and regulatory authorities.

## RISK ASSESSMENT

Risk factors associated with the use of single-use systems may be related to product quality or to the stable supply of biologics. Risks should be identified, and the magnitude of the risk should be estimated using standard risk management practices, which evaluate attributes such as impact and uncertainty, or severity and likelihood. An identified risk should be analysed and compared to risk criteria to decide whether it is acceptable or should be mitigated. The tools that may be used for risk assessment include qualitative techniques such as a risk matrix and semi-quantitative techniques such as risk scores. ICH Q9 is a useful reference for a detailed description of risk assessment.

### Impact on the Quality of Biologics

#### Residual Impurities

One of the quality-related issues resulting from the use of single-use components is the risks of residual impurities including leachables, insoluble particulate matter and insoluble visible matter (hereinafter termed single-use component-related impurities). These impurities could be the critical quality attributes (CQAs) of drug substance/drug product by themselves. In order to avoid potential health hazards, a list of all the impurities that might be introduced into the manufacturing process should be prepared and subjected to risk assessment to identify risks that should be controlled. Information provided by suppliers about the raw materials, manufacturing process, test results and prior experience may be used in addition to the results of tests conducted by end users.

#### Extractables and Leachables

Chemicals derived from single-use components that are introduced into the process solution under conditions of exaggerate temperature, pH or solvent are called extractables, whereas chemicals that migrate under actual process conditions are termed leachables [33–36]. Extractables/leachables may include a variety of chemicals or their derivatives used in the manufacture of single-use components such as catalysts, polymeric initiators, excipients (e.g. antioxidants, lubricants, anti-tack and anti-static agents), oligomers with a low degree of polymerization, adhesives, anchoring agents, adhesive resins and irradiation-induced degradation products and oxides.The chemical profiles of leachables should be identified to the extent possible and feasible on the basis of test results for extractables provided by the supplier, and as necessary by conducting tests on the actual manufacturing process solutions or on simulations, under conditions designed to mimic worst case scenarios. If test results for extractables provided by the supplier are to be used when evaluating leachables, a determination should be made as to whether the extractables preparation procedures and analytical methods are appropriate. Sterilization of single-use components by radiation can also result in additional extractables/leachables; hence, the sterilization conditions have an important role when using test results for extractables provided by the supplier and should be clearly specified. The necessity to test for leachables in either the process solution or a simulation arises when they are difficult to predict from the test results for extractables, or when it is considered possible that a new moiety, may be eluted during the manufacturing process.Risk assessments for all possible leachables should be conducted, and permitted ranges should be defined for chemicals that are considered to require control. Toxicity evaluations should be conducted when analysing and evaluating risk.

#### Insoluble Particulate Matter

**Fig. 1:**
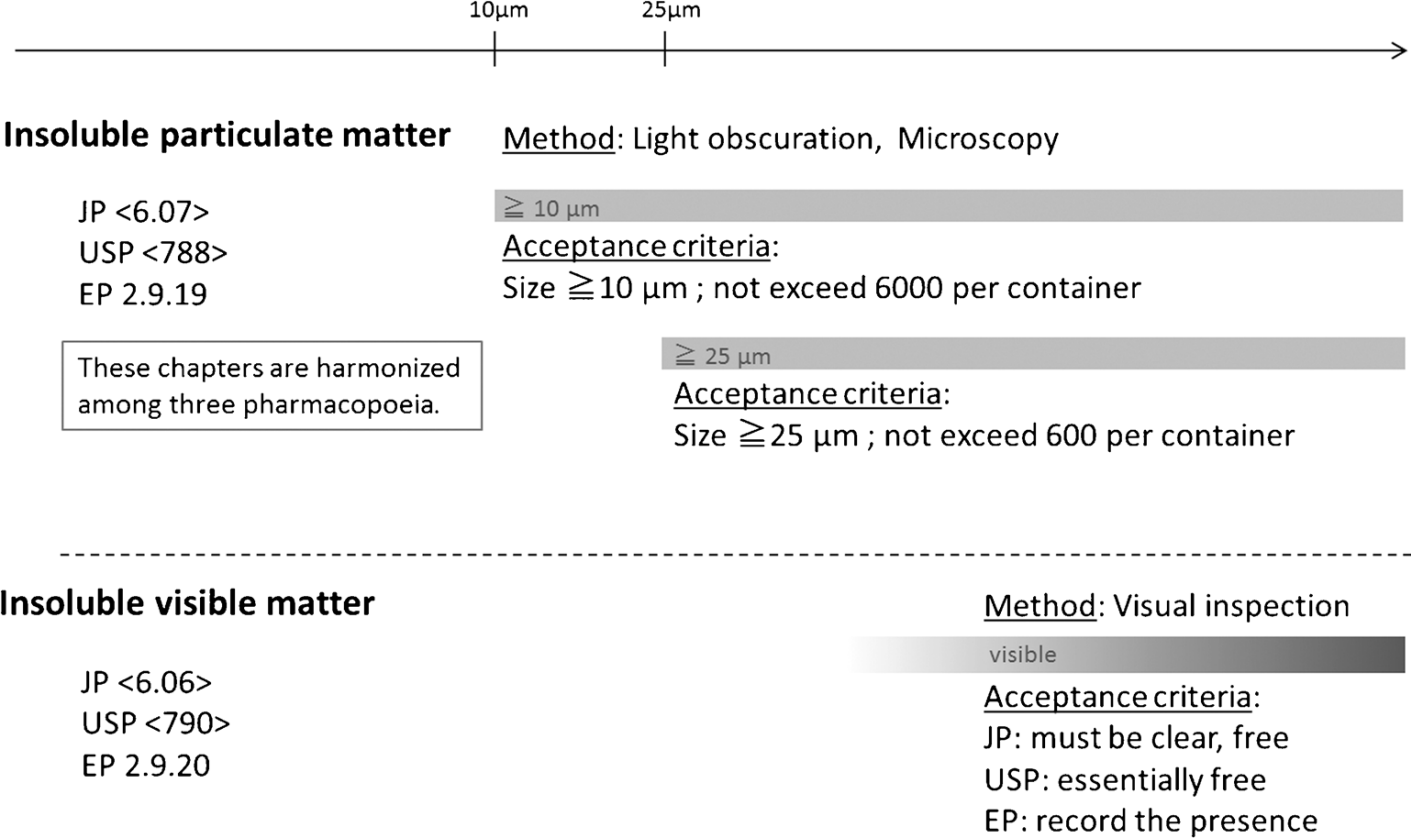
Pharmacopoeia specifications of insoluble particulate matter and insoluble visible matter for injections

In this study, insoluble particulate matter is defined as any invisible insoluble impurity derived from a single-use component and is distinguished from insoluble visible matter. Insoluble particulate matter may derive from an airborne solid in a single-use component manufacturing facility or raw materials or components used in the process of manufacturing the single-use components. There may be various insoluble particulate matters of different composition and particle size.As discussed for the leachables, tests for insoluble particulate matter, generally using water, should be conducted with reference to the information provided by the supplier (e.g. raw material, manufacturing processes, manufacturing environment, procedures to control insoluble particulate matter). Existence and characteristics of insoluble particulate matter derived from single-use components should be identified, to the extent possible and feasible. The *Japanese Pharmacopoeia* ([4] <6.07> “Insoluble particulate matter for injections”), as well as the *European Pharmacopoeia* ([13] <2.9.19> “Particulate contamination: Sub-visible particles”) and the *US Pharmacopoeia* ([9] <788> Particulate matter in injections), adopts the harmonized general chapter and sets the control ranges for insoluble particulate matter of over 10 and 25 μm in diameter (Fig. 1). Accordingly, such insoluble particulate matter in a drug product should be controlled. Risk assessments should be undertaken and control ranges defined for insoluble particulate matter of a size outside the scope of the pharmacopoeia or for new materials not previously detected in drug products, if necessary.

#### Insoluble Visible Matter (Visible Particle)

In this study, insoluble visible matter is defined as any visible impurity adhering to or embedded in the inside (process contact surface) or outside of a single-use component. Insoluble visible matter may be derived from a worker, airborne solids or components present in a manufacturing facility of single use suppliers. If peristaltic pumps are used, insoluble visible matter may be released from the tubing.Each pharmacopoeia lists the requirements for such insoluble visible matter, and drug products must meet these acceptance criteria; (e.g. *Japanese Pharmacopoeia* (JP) <6.06> “Foreign insoluble matter test for injections” [3], *US Pharmacopoeia* (USP) <790> “Visible particulates in injections” [10] and *European Pharmacopoeia* (EP) <2.9.20> “Particulate contamination: Visible particles” [14]). Control ranges for insoluble visible matter in manufacturing processes should be established while bearing in mind that the visible detection limits of insoluble visible matter depend on factors such as the environment in which the visual inspection is conducted and on the transparency of the single-use component.

#### Endotoxins and Microbes

Endotoxins and microorganisms are other impurities that could affect the drug substance/drug product and require control, particularly for injectables, where contamination with endotoxins and microorganisms could negatively impact the health of the patient.

#### Impact on CQAs

An additional quality-related event impacted by the use of single-use components is deviation of the CQAs of biologics from the appropriate limits, range and distribution. Possible deviations include reduced concentration of the contents due to adsorption to the single-use component, changes in aggregate content, changes in the molecular variant content of intended substances such as oxidants and changes in stability.

The magnitude of the risk (impact and uncertainty, or severity and likelihood) arising from the use of single-use components should be estimated for each CQA. Useful factors for estimating the level of risk include the chemical composition of the single-use component, the surface structure, the profile of extractables/leachables, content of endotoxins and microbes, material attributes such as the profile of insoluble particulate matter impurities, the extent of contact between the single-use component and the process (surface area, time, temperature, agitation), the pH and conductivity of the process solution, as well as the operational procedure. The magnitude of the risks relevant to the usage or quality of the single-use component should be compared to the risk criteria, and their acceptability should be justified. In this instance, the control range of CQAs is the risk acceptance criteria and the extent and frequency of deviation from the CQA control range may be considered in risk evaluation.

### Impact on Stable Supply of Biologics

Another event affected by the use of single-use components is the stable supply of biologics. Factors that could impact the stable supply of biologics are the quality and proper usage of single-use components as well as the certainty of their supply chain.

Among the quality attributes of single-use components, the impact of integrity and sterility is potentially large. Leachables from single-use bags for cell culture can have a significant effect on cell growth and metabolism, and deviations in process control can be disruptive, thereby affecting the stable supply of biologics.

As for proper usage of single-use components, factors that could impact the stable supply of biologics include transportation, storage, unpacking, set-up, connection with connectors or other components, as well as the operations in the biologics manufacturing processes and the skill of the operators. In addition to a potential direct effect on manufacturing operations, these factors could also cause leaks, affect the integrity (seal performance) or sterility of single-use components, and indirectly impact stable supply as a result of contamination. Leaks resulting in a contamination of the manufacturing environment and possible exposure of the operator to hazards could also disrupt the stable supply of biologics.

Factors relevant to single-use component quality and usage should be identified, and risk assessments should be conducted to determine the need for relevant controls.

## RISK CONTROL STRATEGIES

Risk control strategies should be determined on the basis of risk assessment to avoid hazards to health, discard or supply interruption of the biologics. For this purpose, it is useful to conduct a risk assessment or more specifically, identification, analysis and evaluate the risks in the manufacturing processes that affect the impurities or other CQA (previous section). Critical process steps and critical process parameters (CPP) should be identified to prioritize the requirements for controls.

Risk controls include various measures such as avoiding, mitigating and removing the source of the risks. One option for averting a health hazard is to avoid using any single-use components whose impact on health or whose leachables are uncertain, until further individual testing is done. Risks leading to out-of-specification results or delay of supply of biologics may be mitigated by judicial selection of appropriate single-use components, by setting their specifications, by carrying out appropriate design, evaluation, and control measures for their use in the manufacturing of biologics. If the combination of the quality control strategies for the single-use components and the manufacturing process of biologics are insufficient to avoid risk, it may be useful to also include the specifications for the drug substance/drug products. It is possible to confirm the validity of established manufacturing processes for biologics through process validation.

When selecting control methods, end users should take the severity, probability and detectability of deviations in process parameters into consideration. In general, it is preferable to establish multiple control methods for each CQA. When establishing control strategies, there should be cooperation between the R&D, process development, manufacturing and procurement departments of the biopharmaceutical industry, in addition to the quality department.

### Selection of Appropriate Single-Use Components

To ensure the quality and stable supply of biologics after considering the abovementioned risks, it is important to choose single-use components suitable for the intended use, for which the certainty of supply and quality is assured. Since these issues primarily depend on controls determined by the supplier, it is important to obtain the suppliers’ relevant quality systems information and to select the single-use components whose quality has been appropriately evaluated and controlled.

The factors that should be taken into consideration include the structural design of the single-use component including the connector joints, raw material controls, manufacturing environment controls, manufacturing process controls, sterilization process controls, test procedures and reliability of results for extractables, test procedures and acceptance criteria for insoluble particulate matter and insoluble visible matter, shipping inspection, packaging/transport controls and change management/notification between the supplier and their sub-suppliers. It is important to select single-use components whose quality has been appropriately controlled, so that differences between individual components or lots do not affect the quality of biologics.

If raw materials such as stearate derived from bovine fat have been used in single-use component manufacturing processes, they should meet the animal-free requirement criteria (e.g. standard for biological ingredients in Japan). It is also critically important to control endotoxins, insoluble particulate matter and insoluble visible matter in single-use components used in the manufacture of drug product (Table I). Decisions to adopt single-use components should be made after taking into account the technical information, including the recommended conditions for use submitted by the supplier, since they may have lower physical strength compared to the materials used in conventional technologies such as stainless-steel components.

**Table I:** Examples of Points to Consider When Introducing Single-Use Components

Examples of lists to be assessed	Examples of issues to be assessed
Structure design	Shape, layered structure for preventing leachables, compatibility with other components, structure for preventing misuse, strength
Raw materials control	Monomers, polymerization initiators, metal catalysts, additives (antioxidants, lubricants, anti-adhesives, antistatics), adhesives, printing inks, solvents, and biological ingredients
Manufacturing environment control	Air cleanliness, airborne microorganisms
Manufacturing process control	Inspections upon receipt of materials and components, shape-forming, film-forming, bag-making, assembly, integrity test
Sterilization process control	Radiation dosage (gamma- and beta-rays), level of ethylene oxide gas
Quality control	Reliability of test procedure and analytical methods for extractables, test procedure and acceptance criteria for insoluble particulate matter
Release test	Appearance, integrity test, consistency with the drawings, endotoxin test, inspection of foreign matters, sterility assurance
Packaging and delivery control	Primary packaging, wrapping, transport

Selection of single-use components should take into account the continuity of the supplier’s business and the existence of any appropriate alternative materials, if necessary. For single-use components fitted with sensors, attention should be paid to the performance of the sensor including the reliability of measurement, and the possibility that sensor constituents may affect the quality of biologics, as well as the possibility that sensor failure may impact the manufacturing process operations.

Where the single-use component is critical for the quality and stable supply of biologics, the supplier should be audited and the quality control systems in place should be checked periodically. It is also useful to prepare a quality contract incorporating such items as product standards/specifications of single-use component, manufacturing processes for single-use components that affect quality standards, record controls, storage conditions applicable to the end user, expiration dates, auditing, change controls, and the means of notifying the end user of changes (Table II).

**Table II:** Examples of Information Normally Disclosed to Users by Suppliers

Organization, equipment, quality control system of the single-use component supplier
• General quality system
• Manufacturing facilities and environment (including manufacturing area, layout of facilities such as warehouses, grade, environmental management)
• Manufacturing supply capacity, delivery time, and supply system
• Procedure to develop or change specifications
• System to qualify the outsourcing contractors such as irradiation sterilization vendors
• Staff education
• Business continuity plan
Methods for manufacturing and quality control • Quality assurance by manufacturing process control (parts identification, foreign material control, connection, integrity test, sterilization, storage method, *etc.*)
• Prevention measures of operational error
• Control methods for foreign material
• List of foreign materials possessed by the supplier
• Control methods of foreign insoluble matter attached to or embedded in single-use components
• Control methods of insoluble particulate matter
• Control methods for endotoxins
• Sterilization procedures and validation
• Qualification of each part of the single-use component
• Compatibility assessment of each part of the single-use component
• Procedure and recording of line clearance (prevention of contamination and wrong assembling, *etc.*)
• Process control, recording, operator education and qualification of assembly
• Release tests (list of tests, methods, acceptance criteria, *etc.*)
• Validation methods for shipping
Test results of single-use component
• Extractables test (test methods including preparation condition of extractables, analytical equipment used, results of analytical method validation, *etc.*)
• Insoluble particulate matter test
• Mechanical strength test
• Gamma-ray irradiation resistance test
• Shelf time test
• Biological safety test
• Oxygen permeability test
• CO_2_ permeability test
• Vapour permeability test
• Solution drainage test
• Test methods for aseptic connection
• Other test required for an assessment of the performance of single-use components
Others • Warranty and expiration dates of the materials
• Storage methods of materials (temperature, humidity, necessity of shielding, *etc.*)
• Contract testing of leachables

### Single-Use Component Specifications/User Requirement Specifications

Specifications for single-use components should be established in cooperation with the supplier, and for critical single-use components, the suitability of each lot should be evaluated. It is also useful to prepare documents listing the specific user requirement specifications for single-use components composed of multiple parts. For example, specifications may be set for insoluble particulate matter, endotoxins and sterility.

### Receipt of Single-Use Components and Their Inspection Before Release for Use

In contrast to fixed equipment processes where the actual equipment is qualified and validated, in single-use systems, it is not possible to use the single-use component identical to the component that have been qualified in actual manufacturing process because each component is used only once. Therefore, it is necessary to check the component upon receipt and before releasing for use, so that differences between individual single-use components or lots do not impact the quality or manufacturability of biologics. Acceptance criteria similar to those applied to raw materials should be defined for the single-use components and applied before release for use. In setting the list of such inspection, the intended usage of single-use components in the biologics manufacturing process and its impact on the quality of biologics should be taken into consideration. Decisions concerning the inspection method and acceptance criteria should preferably be made jointly by the supplier and the end user to ensure maximum conformation to the requirements of the end user. Items that should be checked upon receipt include details of the shipping certificate, product labels, external appearance (visually check for breakage or adherence of foreign matter) and details of any irradiation certificate relevant for sterilization. To the extent possible, it should be confirmed that the materials conform to user requirement specifications and material specifications.

#### Inspection Before Release for Use

It is useful to determine storage conditions and to set inspection before use for single-use component, because breakage or contamination with insoluble visible matter and component interconnections may sometimes be difficult to detect at the time of receipt or may occur later during unpacking or storage. Items that should be checked before use, include the manufacture date (warranty period), storage conditions between receipt and use, conformity to set up and configuration specifications, breakage (e.g. visual inspection) and inspection for the presence of insoluble visible matter.

### Qualification of the Single-Use System

Single-use system components should be evaluated as discussed in “Single-Use Component Specifications/User Requirement Specifications” section, and qualification should be carried out to confirm their suitability for the intended use. In addition, single-use systems in which single-use components are connected to fixed equipment/apparatus should undergo design qualification (DQ), installation qualification (IQ), operational qualification (OQ) and performance qualification (PQ). Single-use systems should also be checked for consistency with the intended design and requirement specifications, and their correct function within the expected operating range should be confirmed.

It should be noted that unlike fixed equipment or apparatus installed in manufacturing facilities, in single-use systems, single-use components identical to the component that have been used in qualification cannot be used in actual manufacturing. Attention should be paid to ensure differences between individual single-use components or lots do not impact the quality and manufacturability of biologics.

### Design, Evaluation and Control of the Manufacturing Process for Biologics

Appropriate design, assessment and control of manufacturing processes of drug substance and drug product can reduce the possibility of deviation of CQAs and minimize contamination by impurities from the single-use components. One approach is to include a strategy for elimination of impurities in the downstream purification processes, although it is better to use single-use components without this concern.

For an identified leachable impacting the biologics quality and requiring control, it is useful to investigate the parameters and processes involved in both the generation and elimination of the leachable, as well as the probability and detectability of deviations of these processes and parameters. Factors that can affect the generation of leachables include the composition of the process solution, the extent of the contact with single-use components (surface area, time, temperature and agitation) and sterilization method such as gamma irradiation, while factors that can affect the elimination of leachables include purification processes such as chromatography and UF/DF process along with their process parameters. Thus, it is possible to control leachables by incorporating steps for their elimination in the design of the manufacturing process of biologics and setting the control range of process parameters and in-process testing. In some cases, it is necessary to flush single-use components before use to reduce the levels of not only residual insoluble particulate matter but also soluble leachables.

When single-use components were used in sterile process, it is also important to establish measures to ensure continued sterility during usage, seal integrity after connection to related process equipment, correct installation and the integrity of the filtration sterilization processes. In order to assure the integrity of the single-use components such as sterilization filters, testing/controls after use may be implemented, as necessary.

The mechanism by which the characteristics of single-use components affect the quality of biologics is not completely understood. Thus, single-use component-related impurities such as leachables and insoluble particulate matter may affect the quality of biologics but be undetected by current analyses. Therefore, in addition to the single-use component quality controls discussed here, consistency of the manufacturing process of biologics is also important.

### Specifications of Biologics

In addition to selecting appropriate single-use components and biologics manufacturing process controls, it is also useful to establish specifications for the drug substance/drug product with respect to residual single-use components-related impurities or other CQAs, if necessary. Defining the specifications prevents the release of lots with out-of-specification with regard to residual impurities or other CQAs.

### Appropriate Training When Using Single-Use Component

Because single-use equipment is often operated manually, human error in unpacking, connecting, installation, and operation of biologics manufacturing processes may affect the stable supply and/or quality of the biologics. To avoid human error, training systems should be implemented and standard operational procedures should be provided to the operators. It is also useful for end users and suppliers to jointly undertake periodic operator training, with the additional aim of upgrading technology, including knowledge and automated systems, as appropriate.

### Communication Between Single-Use Suppliers and End Users

It is preferable for the suppliers to disclose as much information as possible regarding the source of chemicals and the quality controls of the single-use components. If possible, it is also preferable that the end user should disclose the purpose of the single-use components in the biologics manufacturing process to the supplier in order to avoid problems arising from invalidated use. Communication between suppliers and end users can be useful to confirm the appropriateness of selection and usage of the single-use components employed in manufacturing processes of biologics (Tables II and III).

**Table III:** Examples of Information Normally Disclosed to Suppliers by Users

1) Information about solutions that contact single-use components
• General composition
• pH
• Protein concentration
• Volume
• Temperature
• Viscosity
2) Conditions of use of single-use components
• Maximum temperature
• Minimum temperature
• Contact duration
• Use of peristaltic pump
• Maximum pressure
• Use of autoclave sterilization
• Flushing prior to use
• Stirring
• Filter integrity test (sterilization filter)

### Deviation Control

There are known incidents involving single-use components, such as content leakage, although the frequency may be low. Since manual operation is more common with single-use systems than with conventional fixed equipment, technical proficiency, such as in the assembling of components, is necessary to avoid even infrequent incidents such as content leakage. The appropriate responses in cases of deviations, such as leakage or misconnection, should be set out beforehand in accordance with the companies’ established and GMP-compliant deviation handling standard operation procedure (SOP). In the event of a deviation, measures should be taken in accordance with the procedure, and corrective action and preventive action (CAPA) should also be taken, as appropriate and the choice of component should be reevaluated.

## LIFE CYCLE AND CHANGE MANAGEMENT

In the life cycle management of biologics manufactured using single-use systems, it is important to continually assess whether the system employed is being operated as intended and whether improvements or changes are required. In addition, change controls are an important factor in life cycle management even if single-use components are used, because many changes occur in manufacturing processes of biologics due to scale changes in manufacture or implementation of new technologies.

Possible changes at end users of single-use components include a change of suppliers of the single-use components, a change in a single-use component from the same supplier as well as a change in the use of a single-use component. A risk assessment of the impact on the quality of the biologics should be undertaken for each change, in light of information provided by the supplier and the comparability assessed with reference to the ICH Q5E guidelines, as necessary.

Possible changes in the single-use component supplier include changes in the raw materials, parts used, place of manufacture, manufacturing processes, sterilization processes, quality testing, acceptance criteria of release test, packaging and delivery. It is necessary for end users and suppliers to establish in advance what changes might affect the quality of single-use components, with consideration given to an adequate period to investigate the effects of the change. Lot control is required for single-use components as for raw materials and forms part of the control measures imposed on biopharmaceutical industries.

In order to avoid any unexpected deleterious impact on quality attribute due to the changes, suppliers and end users should interact throughout the life cycle of the biologics concerning the quality and purpose for use of the single-use components. An agreement should be clearly established indicating that information relevant to any implemented changes will be provided. Collaboration between suppliers and end users is also recommended in order to ensure a continual improvement of biopharmaceutical quality throughout the biologics life cycle. For such purpose, end users should perform supplier audits periodically to ensure that the suppliers have an appropriate quality system.

## CONCLUSION

Our intention is that this study will be useful in promoting the development of biologics as well as in ensuring their safety, quality and stable supply.

From the international perspective, comments or suggestions on this paper are appreciated. Please send comments by email to “SU_jimukyoku@nihs.go.jp“, if any. Valuable comments will be considered for the future regulatory environment regarding single-use system related issues in Japan.

## GLOSSARY

CQA (critical quality attribute): A physical, chemical, biological, or microbiological property or characteristic that should be within an appropriate limit, range, or distribution to ensure the desired product quality.
Extractables: Chemical entities that are extracted from single-use components into solvents or fluid contents under exaggerate conditions. In this study, specifically, extreme conditions are defined as conditions that are favourable for transfer of chemicals into solvents due to the composition of the solvents, pH, temperature, duration of contact, *etc.*, compared to the general conditions for the manufacturing process of biologics.
Insoluble visible matter: Impurities that are visually observed and insoluble, and attached to or embedded in single-use components.
Insoluble particulate matter: Insoluble particulate impurities that are derived from single-use components.
Leachables: Chemical entities that are extracted from single-use components into the manufacturing process solution under the general conditions of the process.
Process solution: A solution that contacts with single-use components during the manufacturing process of biologics.
Single use: Single use means that the component is used only once in a biopharmaceutical manufacturing process.
Single-use component: A component that is intended to be used once (single use). Specifically, single-use components include plastic bags, filters, tubing, connectors, solution-storage bottles, and sensors.
Single-use system: A system equipped with single-use components.
